# The impact of “4 + 7” volume-based drug procurement on the volume, expenditures, and daily costs of antihypertensive drugs in Shenzhen, China: an interrupted time series analysis

**DOI:** 10.1186/s12913-021-07143-3

**Published:** 2021-11-26

**Authors:** Ying Yang, Ruiwen Tong, Shicheng Yin, Lining Mao, Luxinyi Xu, Siyu Hao, Zongfu Mao

**Affiliations:** 1 School of Health Sciences, Wuhan University, 115# Donghu Road, 430071 Wuhan, China; 2 Global Health Institute, Wuhan University, 115# Donghu Road, 430071 Wuhan, China; 3 Dong Fureng Economic & Social Development School, Wuhan University, 299# Bayi Road, 430072 Wuhan, China

**Keywords:** National centralized drug procurement (NCDP), "4 + 7", Volume-based procurement, Antihypertensive drugs, China

## Abstract

**Background:**

In 2019, Chinese government launched a nationwide volume-based drug procurement aiming at reducing drug prices and saving drug costs through economies of scale, which aroused widespread attention. The first round of the policy pilot was implemented in 4 municipalities and 7 sub-provincial cities, referred to as “4 + 7” policy. In the “4 + 7” policy, 7 antihypertensive drugs were included. This study was conducted to evaluate the impact of “4 + 7” policy on the use of policy-related antihypertensive drugs.

**Method:**

This study applied single-group Interrupted Time Series (ITS) design. We used drug purchasing data from the Centralized Drug Procurement Survey in Shenzhen 2019, covering 24 months from January 2018 to December 2019. Antihypertensive drugs related to “4 + 7” policy were selected as study samples, including 7 drugs in the “4 + 7” List and 17 alternative drugs. Alternative drugs refer to antihypertensive drugs that have an alternative relationship with “4 + 7” List drugs in clinical use and have not yet been covered by the policy. “4 + 7” List drugs were then divided into bid-winning and bid-non-winning products according to the bidding results. Purchase volume, expenditures, and daily costs were selected as outcome variables, and were measured using Defined Daily Doses (DDDs), Chinese Yuan (CNY), and Defined Daily Drug cost (DDDc).

**Results:**

After “4 + 7” policy intervention, the procurement volume of bid-winning antihypertensive drugs significantly increased (3.12 million DDD, 95 % *CI* = 2.14 to 4.10, *p* < 0.001), while the volume of non-winning drugs decreased (-2.33 million DDD, 95 % *CI*= -2.83 to -1.82, *p* < 0.01). The use proportion of bid-winning antihypertensive drugs increased from 12.31 to 87.74 % after policy intervention. The overall costs of the seven “4 + 7” List antihypertensive drugs significantly declined (-5.96 million CNY, 95 % *CI*= -7.87 to -4.04, *p* < 0.001) after policy intervention, with an absolute reduction of 36.37 million CNY compared with the pre-“4 + 7” period. The DDDc of bid-winning antihypertensive drugs significantly decreased (-1.30 CNY, 95 % *CI*= -1.43 to -1.18, *p* < 0.001), while the DDDc of non-winning (0.28 CNY, 95 % *CI* = 0.11 to 0.46, *p* < 0.01) and alternative (0.14 CNY, 95 % *CI* = 0.03 to 0.25, *p* < 0.05) antihypertensive drugs increased markedly.

**Conclusions:**

The implementation of “4 + 7” policy promoted the drug use hypertensive patients gradually concentrated on the quality-guaranteed bid-winning drugs, which might be conducive to improve the overall quality level of drug use of Chinese hypertensive patients. Besides, a preliminary positive policy effect of price cut and cost-saving was observed in the antihypertensive drug category. In the future, price monitoring and drug use management regarding policy-related drugs should also be strengthened.

**Supplementary Information:**

The online version contains supplementary material available at 10.1186/s12913-021-07143-3.

## Introduction

In China, the total health expenditure has grown rapidly from 145.4 billion Chinese Yuan (CNY) in 2008 to 5799.8 billion CNY in 2018, with an average compound annual growth rate of 13.4 % [1]. In 2018, the total drug cost was 218.3 billion CNY in China, accounting for 35.8 % of the total health expenditure, which was much higher than the average level of 17 % in the Organization for Economic Co-operation and Development (OECD) countries [2]. In China, with the increasing aging population, the incidence of hypertension, as well as the overall medication burden of hypertensive patients have increased rapidly year by year [3]. The cardiovascular drug spending accounted for 8.84 % of the total drug spending in China [4]. What’s more, drug costs for hypertensive patients are composed of about 90 % of their outpatient costs [5]. Thus, the price cut and cost saving of antihypertensive drugs have become a noteworthy topic in China.

In January 2019, the General Office of the State Council of the People’s Republic of China (PRC) issued the National Centralized Drug Procurement (NCDP) policy [6]. NCDP is the first policy attempt for nationwide volume-based drug procurement in mainland China, aiming at reducing drug prices and saving drug costs through economies of scale, which aroused widespread attention. In the first round of NCDP pilot, 11 cities were selected as pilot cities including 4 municipalities (Beijing, Tianjin, Shanghai, Chongqing) and 7 sub-provincial cities (Shenyang, Dalian, Xiamen, Guangzhou, Shenzhen, Chengdu, Xi’an), thus known as the “4 + 7” policy. In this policy, 25 drugs (by generic name) in the “4 + 7” procurement list were purchased [7], in which 7 antihypertensive drugs were included, i.e., Amlodipine, Irbesartan, Irbesartan hydrochlorothiazide, Fosinopril, Lisinopril, Losartan, Enalapril. The average price reduction was 59.1 % for the 7 bid-winning antihypertensive products.

Previous studies reported that “4 + 7” policy achieved positive effects in promoting the substitution use of generic drugs against original drugs, reducing drug prices, promoting rational drug use, etc. [8–11]. In terms of the therapeutic category of the 25 drugs in the “4 + 7” procurement list, the impact of the policy might cover patients with a variety of diseases. Hypertension is one of the important chronic diseases with the heaviest disease burden in China, and it is also the disease with the largest number of drugs covered by the 4 + 7 policy, thus the effect of policy on the use of antihypertensive drugs is of great concern. Previous studies have reported relevant explorations. Wang et al.’s survey [12] in the outpatient of a tertiary hospital mentioned that the implementation of “4 + 7” policy increased the use of cardiovascular generic drugs and significantly reduced the drug costs of patients. He et al.’s [11] and Yang et al.’s [13] investigation reminded that the issue of poor efficacy and increased adverse reactions were observed in some ”4 + 7” bid-winning antihypertensive drugs, which may potentially affect the clinical use of related drugs [13, 11]. However, several issues were still unclear, for example, the changes in the consumption structure of bid-winning and bid-non-winning antihypertensive drugs, the utilization of policy covered and uncovered antihypertensive drugs, the potential impacts on hypertensive patients’ medication burden. Thus, we conducted this quasi-natural experiment study to quantitatively evaluate the impact of “4 + 7” policy on the volume, expenditures, and daily cost of policy-related antihypertensive drugs.

## Methods

### The policy intervention

In China, the NCDP policy was initiated and organized by the National Healthcare Security Administration (NHSA) of the PRC. Table 1 illustrates the main timeline of the “4 + 7” policy. The highlight of this policy lies in the implementation of “volume-based procurement”, in which “Trade-for-price” and “Guarantee of use” were taken as the core policy measures [14].

**Table 1:** Timeline for “4 + 7” policy

Date	Key events
June 20, 2018	Premier Li Keqiang presided over a State Council executive meeting to discuss the work of “National Centralized Drug Procurement (NCDP) pilot”.
November 14, 2018	President Xi Jinping presided over the fifth meeting of Commission for Deepening Overall Reform of the CPC Central Committee. This meeting approved the *Pilot Program of National Centralized Procurement Drug Procurement*.
November 15, 2018	Shanghai Joint Procurement Office issued the *4 + 7 City Centralized Drug Procurement Document*, and announced the procurement plan. A total of 31 drug varieties were involved.
December 6, 2018	At 8:30 − 10:00 am, drug manufacturers submit quotation application and relevant materials.
December 6, 2018	At 2:00 pm, carry out negotiations and bidding.
December 7, 2018	Shanghai Joint Purchasing Office announced the proposed bidding results.
December 7, 2018	Chinese government organized the deployment meeting for the pilot work of National Centralized Drug Procurement and Use. Vice premier Sun Chunlan attended the meeting and delivered a speech, emphasized the importance of steady promoting NCDP pilot work.
December 17, 2018	Shanghai Joint Purchasing Office announced the bidding results. 25 drugs (by generic name) in the “4 + 7” List were included, with an average price cut of 52 % and the highest price cut of 96 %.
January 1, 2019	General Office of the State Council issued the *Notice on Issuing the Pilot Program for National Centralized Drug Procurement and Use* (GBF [2019] No.2).
March to April, 2019	From March 1 to April 1, 2019, 11 pilot cities started the procurement of winning products one after another.

- **(a) “Trade-for-price”**: Under the “4 + 7” policy, each public medical institution in the 11 pilot cities was required to submit the annual agreed procurement volume of the “4 + 7” List drugs to the NHSA of the PRC. The agreed procurement volume is the expected annual purchase volume of a certain drug (by generic name) estimated with reference to the use volume in the previous year. The NHSA of the PRC then organized competitive bidding and price negotiation based on the 60 %-70 % of the annual procurement volume of 11 pilot cities. Pharmaceutical manufacturers hold original branded drugs that beyond patent protection period or generic drugs that passed the consistency evaluation of quality and efficacy were eligible to participate in the bidding. The pharmaceutical manufacturer with the lowest bid price won the bid. On December 17, 2018, the Joint Procurement Office announced the list of the 25 bid-winning products (by manufacturer), as well as the bid-winning price [7].
- **(b) “Guarantee of use”**: 11 pilot cities were required to start implementing the bid-winning results before April 1, 2019. The purchases of all the bid-winning products were carried out on the provincial drug bidding and procurement platform. It is worth mentioning that, National Health Commission of the PRC issued a supporting policy that required public medical institutions to give priority to the purchase and use of bid-winning drugs [15]. Besides, the use volume of each bid-winning product in each public medical institution in pilot cities was monitored and assessed by the NHSA of the PRC to ensure the completion of the agreed procurement volume [16]. In most of the pilot cities, medical institutions conduct monthly assessments on relevant clinical departments to ensure the use of bid-winning products.

On March 28, 2019, Shenzhen implemented the “4 + 7” biding results, starting to purchase the 25 bid-winning drugs at the winning bid prices [17].

### Setting and data sources

In this study, the research site is one of the “4 + 7” pilot cities – Shenzhen. Shenzhen is a megacity in South China, and it forms part of the Pearl River Delta megalopolis. Shenzhen consists of 11 districts and 74 subdistricts, with a total administrative area of 1997.47 km^2^ and a total population of 13.44 million in 2019 [18]. In Shenzhen city, the clinical visits of government-run medical institutions were 70.91 million in 2018 and 83.31 million in 2019 [19].

The data used in this study was from the Centralized Drug Procurement Survey in Shenzhen 2019 (CDPS-SZ 2019). The survey was organized and conducted by the Global Health Institute of Wuhan University between December 2019 and January 2020, aiming to evaluate the implementation effect of Shenzhen’s pharmaceutical policy reform. In this survey, we collected the monthly drug purchase order data of medical institutions in Shenzhen between 2017 and 2019, and set up the CDPS-SZ 2019 database. In the CDPS-SZ 2019 database, each purchase order record included purchase date, generic name, dosage form, specification, pharmaceutical manufacturer, price per unit, purchase volume, purchase expenditures, etc. A general database containing 963 127 monthly aggregated purchase order records was established, involving 1079 drug varieties (by generic name), 346 medical institutions, 857 pharmaceutical manufacturers. Details of the CDPS-SZ 2019 are available in the previous work done by our team [9, 20].

In China, under the zero-markup drug policy [21], the drug purchase price in public medical institutions is the same as the prices used by patients. Since 2015, it was required that all drugs used by public medical institutions should be purchased through the provincial-level drug centralized procurement platform [22]. Therefore, in Shenzhen, the drug purchase data of public medical institutions in the CDPS-SZ 2019 database is generally consistent with the drug use data.

### Samples

In this study, we extracted data from the CDPS-SZ 2019 database with the following criteria: (a) the drug scope was “4 + 7” policy-related antihypertensive drugs, including 7 antihypertensive drugs in the “4 + 7” procurement list [23] and the alternative antihypertensive drugs that have an alternative relationship with “4 + 7” List drugs in clinical use (Supplementary Table 1). The alternative antihypertensive drugs were determined based on the *Monitoring Plan Work of National Centralized Drug Procurement and Use* issued by the NHSA of the PRC [16]. The “4 + 7” List drugs were then sorted into bid-winning products and non-winning products [7]. Bid-winning products referred to products that won the tender in “4 + 7” policy, otherwise they were deemed to be non-winning products. (b) the time period was 24 months from January 2018 and December 2019. (c) the scope of medical institutions were all the public medical institutions in Shenzhen. In China, public medical institution refers to government-run non-profit medical institution, including government-run hospitals, community healthcare centers, etc. [24]

Purchase order records with incomplete information were excluded. Finally, 18,115 purchase order records of 24 antihypertensive drugs (by generic name) were included in the analysis.

### Outcome measures

Three outcome measures were included: volume, expenditures, and daily cost. Expenditure data was reported in CNY. Volume was measured using Defined Daily Doses (DDDs), a measurement developed by the World Health Organization (WHO) to compare drug consumptions [25]. In this study, the DDD value of each medication is determined according to the *Guidelines for ATC classification and DDD assignment 2021* [26]. Daily cost of each group of drugs was assessed by Defined Daily Drug cost (DDDc), which was calculated as *Expenditures*/*DDDs*.

### Statistical analysis

**Fig. 1:**
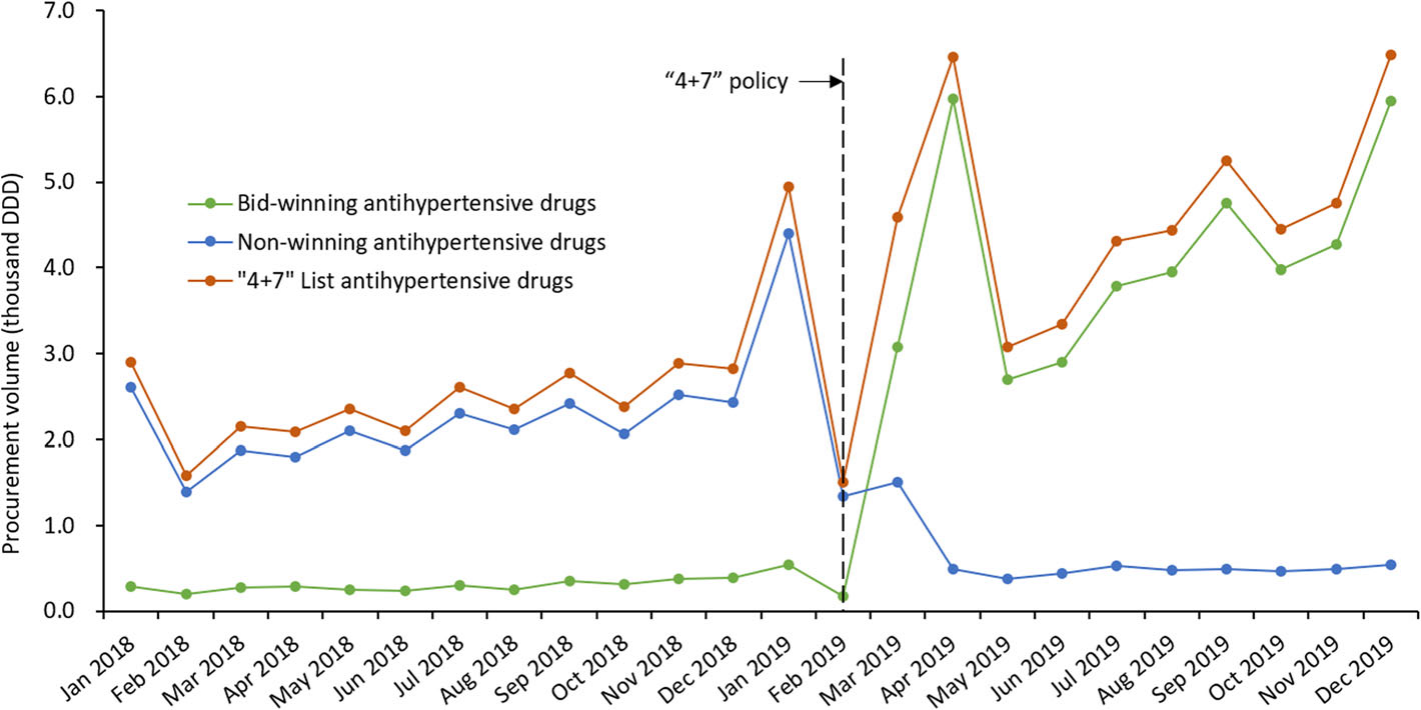
Monthly trends of procurement volume of bid-winning and non-winning antihypertensive drugs between January 2018 and December 2019.

Descriptive statistics were used. We first described the volume, expenditures, and DDDc of policy-related antihypertensive drugs before and after the implementation of “4 + 7” policy. Considering the seasonality of drugs use [27], which is also apparently outlined in Fig. 1, we compared the corresponding period before (March to December 2018) and after (March to December 2019) the policy intervention in the descriptive analysis.

A single-group Interrupted Time Series (ITS) was designed to assess the change of volume, expenditures, and DDDc associated with the implementation of “4 + 7” policy. ITS is a commonly used approach for evaluating changes in longitudinal series following a quasi-experimental intervention occurring at a fixed point in time [28]. The data of 24 months from January 2018 and December 2019 were used. The date of implementing “4 + 7” bid-winning results in Shenzhen (March 2019) was regarded as the intervention time point for ITS analyses. We used segmented regression models that control for baseline trends to estimate changes in the levels and trends of each outcome variable after “4 + 7” policy. The following model was developed [29]:

$${Y}_{t}={\beta }_{0}+{\beta }_{1}\times {time}_{t}+{\beta }_{2}\times {intervention}_{t}+{\beta }_{3}\times time after {intervention}_{t}+{\epsilon }_{t}$$

*Y*_*t*_ is the outcome variable (volume, expenditures, and DDDc). *β*_*0*_ estimates the baseline level of the outcome variable at the beginning of the observation period. *β*_*1*_ estimates the slope prior to intervention. *β*_*2*_ estimates the change in level in the period immediately following policy intervention. *β*_*3*_ estimates the differences between pre- and post-intervention slopes. *ε*_*t*_ is an estimate of the random error at time *t*. We used the Cumby-Huizinga test to detect the autocorrelation of each model [30]. If auto-correlation is detected, the Prais-Winsten method was applied to estimate the regression [31]. The Durbin–Watson test was performed to indicate the model’s adjustment effect on autocorrelation, a Durbin–Watson *d* value of around 2 indicates no sign of auto-correlation [32]. All analyses were performed using Stata version 16.0.

## Results

### Descriptive analysis

A total of 24 antihypertensive drugs (by generic name) purchased between January 2018 and December 2019 were included in this study. Among them, 7 were “4 + 7” List drugs and 17 were alternative drugs. A total of 36 pharmaceutical manufactures and 71 public medical institutions were involved. The total purchase volume and expenditures of included antihypertensive drugs were 212.03 million DDD and 545.92 million CNY, respectively. As shown in Table 2, the time to market of seven bid-winning antihypertensive drugs in mainland China ranges from December 2017 to November 2018, the bid-winning prices are between 0.20 to1.09 CNY per pill with the price drop of 60.27 %-82.66 % when compared with the average price before the policy.

**Table 2:** General characteristics of bid-winning antihypertensive drugs

Generic name	Dosage form	Specifications	Bid-winning enterprises	Time to market	Bid-winning prices (CNY per pill)	Prices before the policy (CNY per pill)	Price drop (%)
Irbesartan	tablet	75 mg	Zhejiang Huahai Pharmaceutical Co., Ltd.	Dec. 2017	0.20	0.66	-69.41
Amlodipine	tablet	5 mg	Zhejiang Jingxin Pharmaceutical Co.,Ltd.	Nov. 2018	0.15	-	-
Fosinopril	tablet	10 mg	Sino-American Shanghai Squibb Pharmaceuticals Ltd.*	Dec. 2017	0.84	2.79	-69.82
Irbesartan hydrochlorothiazide	tablet	150 mg + 12.5 mg	Zhejiang Huahai Pharmaceutical Co., Ltd.	Dec. 2017	1.09	3.72	-70.71
Lisinopril	tablet	10 mg	Zhejiang Huahai Pharmaceutical Co., Ltd.	Dec. 2017	0.23	1.33	-82.66
Losartan	tablet	50 mg	Zhejiang Huahai Pharmaceutical Co., Ltd.	Dec. 2017	1.05	2.64	-60.27
Enalapril	tablet	5 mg	Yangtze River Pharmaceutical Group Co.,Ltd.	Apr. 2018	0.56	1.88	-70.33

Note. * original drug

The monthly trend chart of procurement volume showed that (Fig. 1), the volume of bid-winning antihypertensive drugs increased, while that of bid-non-winning drugs decreased sharply.

Table 3 shows the changes in volume, expenditures, and DDDc of policy-related antihypertensive drugs in the corresponding period before (March to December 2018) and after (March to December 2019) the implementation of “4 + 7” policy. After policy intervention, the bid-winning antihypertensive drugs increased markedly in the volume (1268.54 %) and expenditures (206.42 %), and decreased in the DDDc (77.61 %). Non-winning antihypertensive drugs reduced by 73.11 % and 70.93 % in the volume and expenditures, and increased by 8.11 % in the DDDc. As for the alternative drugs that have an alternative relationship with “4 + 7” List drugs in clinical use, the volume and expenditures increased by 25.04 % and 23.12 %, and the DDDc slightly dropped by 1.53 %. In terms of the overall policy-related antihypertensive drugs included in this study, the volume raised by 47.63 %, the expenditures and DDDc decreased by 0.63 % and 32.69 %. As for the use proportion of drugs between bid-winning and bid-non-winning drugs, bid-winning drugs increased from 12.31 to 87.74 % after policy intervention; non-winning drugs decreased from 87.69 to 12.26 %.

**Table 3:** The change of volume, expenditures, and DDDc of policy-related antihypertensive drugs in the pre- and post-“4 + 7” periods

	Volume (thousand DDD)			Expenditures (thousand CNY)			DDDc (CNY)		
	Pre-	Post-	GR (%)	Pre-	Post-	GR (%)	Pre-	Post-	GR (%)
“4 + 7” List drugs	24.53	47.13	92.15	73.81	37.44	-49.28	3.01	0.79	-73.60
Bid-winning drugs	3.02	41.35	1268.54	5.76	17.66	206.42	1.91	0.43	-77.61
Non-winning drugs	21.51	5.78	-73.11	68.05	19.78	-70.93	3.16	3.42	8.11
Alternative drugs	48.36	60.46	25.04	151.16	186.11	23.12	3.13	3.08	-1.53
Overall policy-related	72.89	107.60	47.63	224.97	223.55	-0.63	3.09	2.08	-32.69

DDD, Daily Defined Dose; CNY, Chinese Yuan; DDDc, Defined Daily Drug cost; GR, Growth Rate. Pre- refers to March to December 2018; Post- refers to March to December 2019

Table 4 illustrates the changes of each of the seven “4 + 7” List drug. After the implementation of “4 + 7” policy, 7 antihypertensive drugs in the “4 + 7” List increased by 22.60 million DDD (92.15 %) in the volume, and decreased by 36.37 million CNY (-49.28 %) and 2.22 CNY (-73.60 %) in the expenditures and DDDc, respectively. The volume of 7 drugs all increased compared with the pre-“4 + 7” period, with the growth rate ranging from 14.69 to 123.10 %. Five of the 7 drugs decreased in the expenditures after “4 + 7” policy, with the reduction ranging from 16.47 to 60.95 %. As for the DDDc of “4 + 7” List antihypertensive drugs, Enalapril increased by 42.61 %, and five drugs decreased between 54.81 % and 82.50 %.

**Table 4:** The change of the volume, expenditures, and DDDc of each “4 + 7” List antihypertensive drug in the pre- and post-“4 + 7” periods

	Volume (million DDD)			Expenditures (million CNY)			DDDc (CNY)		
	Pre-	Post-	GR (%)	Pre-	Post-	GR (%)	Pre-	Post-	GR (%)
Amlodipine	11790.55	26305.12	123.10	39505.03	15424.75	-60.95	3.35	0.59	-82.50
Irbesartan	6017.09	9736.77	61.82	15173.01	7709.53	-49.19	2.52	0.79	-68.60
Irbesartan hydrochlorothiazide	1909.19	3899.69	104.26	6639.64	5545.92	-16.47	3.48	1.42	-59.11
Fosinopril	572.97	657.15	14.69	3081.37	1531.17	-50.31	5.38	2.33	-56.67
Lisinopril	0.00	6.02	-	0.00	2.77	-	-	0.46	-
Losartan	3339.10	4749.23	42.23	8872.47	5702.14	-35.73	2.66	1.20	-54.81
Enalapril	900.38	1780.08	97.70	540.23	1523.16	181.95	0.60	0.86	42.61
Total	24529.28	47134.07	92.15	73811.75	37439.44	-49.28	3.01	0.79	-73.60

DDD, Daily Defined Dose; CNY, Chinese Yuan; DDDc, Defined Daily Drug cost; GR, Growth Rate. Pre- refers to March to December 2018; Post- refers to March to December 2019

### ITS analysis

The results of ITS analysis (Table 5) indicated that the volume of bid-winning antihypertensive drugs significantly increased by 3.12 million DDD (95 % *CI* = 2.14 to 4.10, *p* < 0.001) after “4 + 7” policy. The volume of non-winning antihypertensive drugs significantly decreased 2.33 million DDD (95 % *CI* = -2.83 to -1.82, *p* < 0.001), and the slope significantly decreased in the post-intervention period (-0.11 million DDD per month, 95 % *CI* = -0.19 to -0.03, *p* < 0.01). No significant differences were detected for the volume of “4 + 7” List drugs, alternative drugs, and the overall policy-related antihypertensive drugs (all *p*-values > 0.05).

**Table 5:** Results of ITS analysis for the change of volume, expenditures, and DDDc of policy-related antihypertensive drugs

	Volume		Expenditures		DDDc	
	*Coef.*	95 % *CI*	*Coef.*	95 % *CI*	*Coef.*	95 % *CI*
**Bid-winning drugs**						
Baseline change, β_0_	0.24	(-0.35, 0.83)	0.47	(0.18, 0.77)**	2.04	(1.96, 2.12)***
Baseline trend, β_1_	0.01	(-0.07, 0.09)	0.02	(-0.02, 0.06)	-0.02	(-0.03, -0.01)**
Level change, β_2_	3.12	(2.14, 4.10)***	0.93	(0.45, 1.42)**	-1.30	(-1.43, -1.18)***
Trend change, β_3_	0.13	(-0.02, 0.28)	0.01	(-0.06, 0.09)	0.01	(-0.01, 0.03)
*R*^*2*^	0.94		0.85		0.99	
Durbin-Watson *d*	2.12		2.11		1.91	
**Non-winning drugs**						
Baseline change, β_0_	1.66	(1.36, 1.96)***	5.39	(4.40, 6.38)***	3.20	(3.09, 3.30)***
Baseline trend, β_1_	0.09	(0.05, 0.13)***	0.26	(0.13, 0.39)***	-0.004	(-0.02, 0.01)
Level change, β_2_	-2.33	(-2.83, -1.82)***	-6.92	(-8.57, -5.28)***	0.28	(0.11, 0.46)**
Trend change, β_3_	-0.11	(-0.19, -0.03)**	-0.34	(-0.59, -0.09)*	0.01	(-0.02, 0.03)
*R*^*2*^	0.92		0.91		0.65	
Durbin-Watson *d*	2.20		2.11		2.00	
**“4 + 7” List drugs**						
Baseline change, β_0_	1.97	(1.19, 2.74)***	5.88	(4.73, 7.02)***	3.06	(2.89, 3.24)***
Baseline trend, β_1_	0.09	(-0.01, 0.19)	0.28	(0.13, 0.43)**	-0.01	(-0.03, 0.02)
Level change, β_2_	0.95	(-0.33, 2.23)	-5.96	(-7.87, -4.04)***	-1.94	(-2.21, -1.66)***
Trend change, β_3_	0.02	(-0.18, 0.22)	-0.33	(-0.62, -0.04)*	-0.04	(-0.09, 0.002)
*R*^*2*^	0.74		0.84		0.98	
Durbin-Watson *d*	2.15		2.15		1.96	
**Alternative drugs**						
Baseline change, β_0_	3.91	(3.11, 4.71)***	12.76	(10.36, 15.17)***	3.24	(3.18, 3.31)***
Baseline trend, β_1_	0.16	(0.05, 0.26)**	0.41	(0.10, 0.73)*	-0.02	(-0.02, -0.01)**
Level change, β_2_	-0.91	(-2.24, 0.42)	-1.99	(-5.99, 2.02)	0.14	(0.03, 0.25)*
Trend change, β_3_	0.01	(-0.20, 0.21)	-0.01	(-0.62, 0.60)	0.00	(-0.02, 0.01)
*R*^*2*^	0.62		0.56		0.59	
Durbin-Watson *d*	2.25		2.18		2.03	
**Overall policy-related** **antihypertensive drugs**						
Baseline change, β_0_	5.85	(4.49, 7.20)***	18.59	(15.28, 21.90)***	3.18	(3.09, 3.27)***
Baseline trend, β_1_	0.25	(0.07, 0.43)**	0.70	(0.26, 1.13)**	-0.01	(-0.02, 0.00)*
Level change, β_2_	-0.01	(-2.26, 2.25)	-8.02	(-13.54, -2.51)**	-0.78	(-0.92, -0.63)***
Trend change, β_3_	0.02	(-0.33, 0.36)	-0.35	(-1.19, 0.49)	-0.02	(-0.04, 0.004)
*R*^*2*^	0.74		0.47		0.98	
Durbin-Watson *d*	2.36		2.24		2.35	

DDDc, Defined Daily Drug cost; *CI*, confidence interval

ITS analysis indicated that the expenditures of bid-winning antihypertensive drugs significantly increased by 0.93 million CNY (95 % *CI* = 0.45 to 1.42, *p* < 0.01) after “4 + 7” policy. The expenditures of non-winning antihypertensive drugs significantly decreased by 6.92 million CNY (95 % *CI* = -8.57 to -5.28, *p* < 0.001), and the slope significantly declined in the post-intervention period (-0.34 million CNY per month, 95 % *CI* = -0.59 to -0.09, *p* < 0.05). The expenditures of “4 + 7” List antihypertensive drugs significantly decreased by 5.96 million CNY (95 % *CI* = -7.87 to -4.04, *p* < 0.001), and the slope significantly decreased in the post-intervention period (-0.33 million CNY per month, 95 % *CI* = -0.62 to -0.04, *p* < 0.05). No significant difference was observed for the alternative drugs neither in expenditure level change nor in expenditure trend change (all *p*-values > 0.05). After the implementation of “4 + 7” policy, the expenditures of the overall policy-related antihypertensive drugs significantly decreased by 8.02 million CNY (95 % *CI* = -13.54 to -2.51, *p* < 0.01).

After policy intervention, the prominent decline of DDDc were observed for bid-winning antihypertensive drugs (-1.30 CNY, 95 % *CI* = -1.43 to -1.18, *p* < 0.001), “4 + 7” List antihypertensive drugs (-1.94 CNY, 95 % *CI* = -2.21 to -1.66, *p* < 0.001), and the overall policy-related antihypertensive drugs (-0.78 CNY, 95 % *CI* = -0.92 to -0.63, *p* < 0.001). The DDDc of non-winning antihypertensive drugs (0.28 CNY, 95 % *CI* = 0.11 to 0.46, *p* < 0.01) and alternative drugs (0.14 CNY, 95 % *CI* = 0.03 to 0.25, p < 0.05) significantly increased by 8.82 % and 4.26 %.

## Discussion

In the present study, we analyzed the impact of the “4 + 7” policy on the volume, expenditures, and DDDc of policy-related antihypertensive drugs. After the implementation of “4 + 7” policy, the DDDc of bid-winning antihypertensive drugs decreased remarkably, as well as the “4 + 7” List drugs and the overall policy-related antihypertensive drugs, which indicated the potential policy effect of relieving medication burden of hypertensive patients. In this study, the ITS analysis indicated a prominent increase in the DDDc of bid-non-winning antihypertensive drugs and the alternative antihypertensive drugs. Our finding fits well with Wang et al.’s [8] study on the overall “4 + 7” policy-related drugs in nine “4 + 7” pilot cities and 12 non-pilot provinces. We supposed there is certain possibility that the price of drugs without policy intervention have increased after policy implementation. The increasing of DDDc for the non-winning and the alternative drugs might be related to the unreasonable prescription behavior, such as increased daily doses of some antihypertensive drugs [33, 34]. In the future, it is recommended to strengthen the price monitoring and prescription management of NCDP policy-related drugs.

Besides, we found that there were significant differences in the changes of purchase volume for different drugs (by generic name) before and after policy intervention, and the growth rate of purchase volume fluctuated between 14.69 % and 123.10 %. This may be related to their differences in time to market, patient loyalty, efficacy differences with original drug, etc. Previous studies reported that some bid-winning antihypertensive drugs might have poor efficacy and increased adverse reactions [11, 13], which attracted widespread concern. However, existing real-world evidence also indicates that 14 bid-winning generic drugs were equivalent to the original drugs in clinical efficacy and use, with no significant difference [35]. Thus, we recommended that relevant investigation at the patient-level might still be necessary to deeply specifically understand the therapeutic effect and patient satisfaction of each drug in the NCDP procurement list.

The present study found that, on the one hand, the consumption of “4 + 7” List antihypertensive drugs (the integration of bid-winning and non-winning drugs) increased 22.6 thousand DDDs (92.15 % increment), which should be attributed to the significant increase in the use of bid-winning antihypertensive drugs. This is closely related to the significant price reduction of bid-winning antihypertensive drugs, and thus implied that the affordability of antihypertensive drugs covered by the policy increased significantly for Chinese patients [12]. On the other hand, the utilization of bid-non-winning antihypertensive drugs decreased by 15.73 thousand DDDs, and this part of the demand also transferred to the bid-winning drugs. These fits with previous findings regarding the impact of “4 + 7” policy on antidepressants [36], anti-hepatitis B virus drugs [37], and antibiotics [20]. After policy intervention, the clinical use of antihypertensive drugs covered by the “4 + 7” procurement list is dominated by the bid-winning products in the setting of public medical institutions.

According to the procurement rules of NCDP policy, only generic drugs that have passed the consistency evaluation of quality and efficacy or original drugs were eligible to participate in the nationwide volume-based procurement [14]. In other words, the bid-winning products in “4 + 7” policy are all quality-guaranteed drugs. Therefore, after the implementation of “4 + 7” policy, the drug use of hypertensive patients gradually concentrate on the bid-winning products, which will be conducive to improve the overall quality level of drug use among Chinese hypertensive patients. In the first round of NCDP policy, only seven antihypertensive drugs were involved, thus the policy effect on improving the quality level of drug use might still be limited. As of August 2021, NCDP policy has reached the fifth round, a total of 219 drugs (by generic name) were involved, in which 30 were cardiovascular system drugs [38]. We can expect that, in the near future, China’s NCDP policy might benefit patients on a larger scale.

After the implementation of “4 + 7” policy, the costs of the seven “4 + 7” List antihypertensive drugs prominently decreased by 49.28 % (36.37 million CNY) compared with the pre-“4 + 7” period. It indicated that there is a initiatory cost-saving effect of “4 + 7” policy on the category of antihypertensive drugs. Price is the primary determinant of drug affordability [39], the unreasonable price and high expenditures of antihypertensive drugs have been an important factor restricted the effective control of medication burden of hypertensive patients in China [40]. Gheorghe et al. [41] reported that the annual medical expenditures per capita of hypertensive patients in low- and middle-income countries exceeds 5.9 times total medical expenditures per capita. Wang et al.’s [42] survey in Gansu, China indicated that the average monthly drug expenditures of hypertensive patients accounted for 16.4 % of the total family expenses. Under the circumstance, “4 + 7” policy has achieved initiatory cost-saving effects in the short term by including 7 antihypertensive drugs. In the future, more commonly used drugs such as medication for chronic diseases or drugs with large demand will be included in the volume-based procurement catalog, which is expected to effectively reduce the drug burden of patients in China.

## Limitations

Several potential limitations should be mentioned regarding the present study. First, “4 + 7” policy was implemented in 11 pilot cities, while limited by the data accessibility, this study only involved one pilot city (Shenzhen) for analysis. Second, the results of this study were based on drug purchase data, rather than drug use data (such as prescriptions). Although there is strong consistency between purchase data and use data under a series of policies. There is still a possibility that the two data sources may not exactly match, so there are certain limitations. Third, this study constructed ITS models using the data of 24 months and only 10 time points after policy intervention were involved, which may have certain limitations in exploring the long-term trend of “4 + 7” policy.

## Conclusions

This study assessed the impact of “4 + 7” policy on policy-related hypertensive drugs in Shenzhen, China. After policy intervention, the consumption of “4 + 7” List antihypertensive drugs increased significantly (92.15 % increment) in the setting of Shenzhen public medical institutions, which attributed to the increased use of bid-winning antihypertensive drugs (1268.54 % increment). Besides, the use proportion of bid-non-winning drugs significantly decreased and the consumption demands of non-winning drugs transferred to bid-winning drugs, which might be conducive to improve the overall quality level of drug use among Chinese hypertensive patients. The DDDc of bid-winning antihypertensive drugs significantly decreased by 63.79 %, the overall costs of the 7 antihypertensive drugs in the “4 + 7” List declined prominently (49.28 %). A preliminary positive policy effect of price cut and cost-saving was observed in the antihypertensive drug category, indicated the potential benefits of “4 + 7” policy on hypertensive patients. However, the DDDc of non-winning hypertensive drugs and alternative drugs increased after policy intervention, suggesting that the price monitoring and drug use management regarding NCDP policy-related drugs should be strengthened in the future.

## Supplementary information

**Additional file 1**

## Data Availability

The data used in this study is not publicly available. The dataset (CDPS-SZ 2019 database) used and analyzed during the current study is available from the corresponding author on reasonable request. In this study, no secondary data is used and no humans are involved.

## Abbreviations

CNY: Chinese Yuan
OECD: Organization for Economic Co-operation and Development
PRC: People’s Republic of China
NCDP: National Centralized Drug Procurement
NHSA: National Healthcare Security Administration
CDPS-SZ 2019: Centralized Drug Procurement Survey in Shenzhen 2019
DDDs: Defined Daily Doses
WHO: World Health Organization
DDDc: Defined Daily Drug cost
ITS: Interrupted Time Series
