# Supplementary material for: The impact of “4 + 7” volume-based drug procurement on the volume, expenditures, and daily costs of antihypertensive drugs in Shenzhen, China: an interrupted time series analysis
Source: BMC Health Serv Res. 2021 Nov 26;21:1275. doi: 10.1186/s12913-021-07143-3 (PMC8620621; doi:10.1186/s12913-021-07143-3)
Supplement: Supplementary file 1 — Additional file 1 [file 12913_2021_7143_MOESM1_ESM.zip › SupplementaryTables.docx]

# Supplementary table 1. The list of included drugs.

| Categories | Generic name |
| --- | --- |
| “4+7” List drugs | Amlodipine, Irbesartan, Irbesartan hydrochlorothiazide, Fosinopril, Lisinopril, Losartan, Enalapril |
| Alternative drugs | Amlodipine Atorvastatin, Felodipine, Lassidipine, Nicardipine, Nitrendipine, Nifedipine, Levoamlodipine, Benazepril, Captopril, Candesartan cilexetil, Losartan Hydrochlorothiazide, Perindopril, Perindopril indapamide, Telmisartan, Valsartan, Valsartan and Amlodipine, Enalapril Folic Acid |

# Supplementary table 2. The results of Cumby-Huizinga test.

|  | Volume | | |  | Expenditures | | |  | DDDc | | |
| --- | --- | --- | --- | --- | --- | --- | --- | --- | --- | --- | --- |
|  | lag | chi2 | p-value |  | lag | chi2 | p-value |  | lag | chi2 | p-value |
| Bid-winning drugs | 1 | 1.751 | 0.186 |  | 1 | 0.924 | 0.337 |  | 1 | 0.015 | 0.902 |
|  | 2 | 1.912 | 0.167 |  | 2 | 5.163 | 0.023 |  | 2 | 0.027 | 0.869 |
|  | 3 | 0.192 | 0.661 |  | 3 | 0.311 | 0.577 |  | 3 | 0.283 | 0.595 |
|  | 4 | 0.057 | 0.812 |  | 4 | 0.283 | 0.595 |  | 4 | 1.525 | 0.217 |
|  | 5 | 0.599 | 0.439 |  | 5 | 0.143 | 0.705 |  | 5 | 1.582 | 0.208 |
|  | 6 | 1.137 | 0.286 |  | 6 | 0.948 | 0.330 |  | 6 | 0.511 | 0.475 |
| Non-winning drugs | 1 | 8.778 | 0.003 |  | 1 | 7.568 | 0.006 |  | 1 | 0.017 | 0.897 |
|  | 2 | 1.278 | 0.258 |  | 2 | 1.049 | 0.306 |  | 2 | 0.202 | 0.653 |
|  | 3 | 0.393 | 0.531 |  | 3 | 0.493 | 0.483 |  | 3 | 0.199 | 0.655 |
|  | 4 | 0.103 | 0.748 |  | 4 | 0.080 | 0.778 |  | 4 | 6.094 | 0.014 |
|  | 5 | 0.460 | 0.498 |  | 5 | 0.560 | 0.454 |  | 5 | 0.240 | 0.624 |
|  | 6 | 0.112 | 0.738 |  | 6 | 0.209 | 0.648 |  | 6 | 0.566 | 0.452 |
| “4+7” List drugs | 1 | 1.985 | 0.159 |  | 1 | 6.471 | 0.011 |  | 1 | 0.116 | 0.734 |
|  | 2 | 1.787 | 0.181 |  | 2 | 0.471 | 0.493 |  | 2 | 0.925 | 0.336 |
|  | 3 | 1.493 | 0.222 |  | 3 | 0.115 | 0.734 |  | 3 | 0.170 | 0.680 |
|  | 4 | 0.041 | 0.839 |  | 4 | 0.050 | 0.822 |  | 4 | 4.277 | 0.039 |
|  | 5 | 0.122 | 0.727 |  | 5 | 0.722 | 0.396 |  | 5 | 0.602 | 0.438 |
|  | 6 | 0.299 | 0.585 |  | 6 | 0.100 | 0.752 |  | 6 | 0.028 | 0.868 |
| Alternative drugs | 1 | 4.695 | 0.030 |  | 1 | 4.200 | 0.040 |  | 1 | 0.249 | 0.618 |
|  | 2 | 0.268 | 0.605 |  | 2 | 0.191 | 0.662 |  | 2 | 0.460 | 0.498 |
|  | 3 | 0.000 | 0.987 |  | 3 | 0.000 | 0.984 |  | 3 | 1.603 | 0.205 |
|  | 4 | 0.156 | 0.693 |  | 4 | 0.058 | 0.811 |  | 4 | 0.744 | 0.388 |
|  | 5 | 0.173 | 0.677 |  | 5 | 0.086 | 0.770 |  | 5 | 1.919 | 0.166 |
|  | 6 | 0.072 | 0.788 |  | 6 | 0.004 | 0.948 |  | 6 | 0.025 | 0.874 |
| Overall policy-related antihypertensive drugs | 1 | 4.518 | 0.034 |  | 1 | 5.747 | 0.017 |  | 1 | 2.978 | 0.084 |
|  | 2 | 0.864 | 0.353 |  | 2 | 0.000 | 0.990 |  | 2 | 2.257 | 0.133 |
|  | 3 | 0.832 | 0.362 |  | 3 | 0.003 | 0.959 |  | 3 | 2.256 | 0.133 |
|  | 4 | 0.032 | 0.859 |  | 4 | 0.085 | 0.770 |  | 4 | 0.439 | 0.508 |
|  | 5 | 0.143 | 0.705 |  | 5 | 0.229 | 0.632 |  | 5 | 0.158 | 0.692 |
|  | 6 | 0.017 | 0.896 |  | 6 | 0.045 | 0.831 |  | 6 | 0.637 | 0.425 |
